# Correction: Removal of Pb(ii) and Cd(ii) from wastewater using arginine cross-linked chitosan–carboxymethyl cellulose beads as green adsorbent

**DOI:** 10.1039/c9ra90094b

**Published:** 2020-01-15

**Authors:** Kaiser Manzoor, Mudasir Ahmad, Suhail Ahmad, Saiqa Ikram

**Affiliations:** Biopolymer Research Laboratory, Department of Chemistry, Jamia Millia Islamia New Delhi India sikram@jmi.ac.in; Applied Chemistry, School of Natural & Applied Science, Northwestern Polytechnical University P. R. China

## Abstract

Correction for ‘Removal of Pb(ii) and Cd(ii) from wastewater using arginine cross-linked chitosan–carboxymethyl cellulose beads as green adsorbent’ by Kaiser Manzoor *et al.*, *RSC Adv.*, 2019, **9**, 7890–7902.

The authors regret that an incorrect version of the SEM image shown in Fig. 3b was included in the original article. The correct version of Fig. 3 is presented below.

**Fig. 3:**
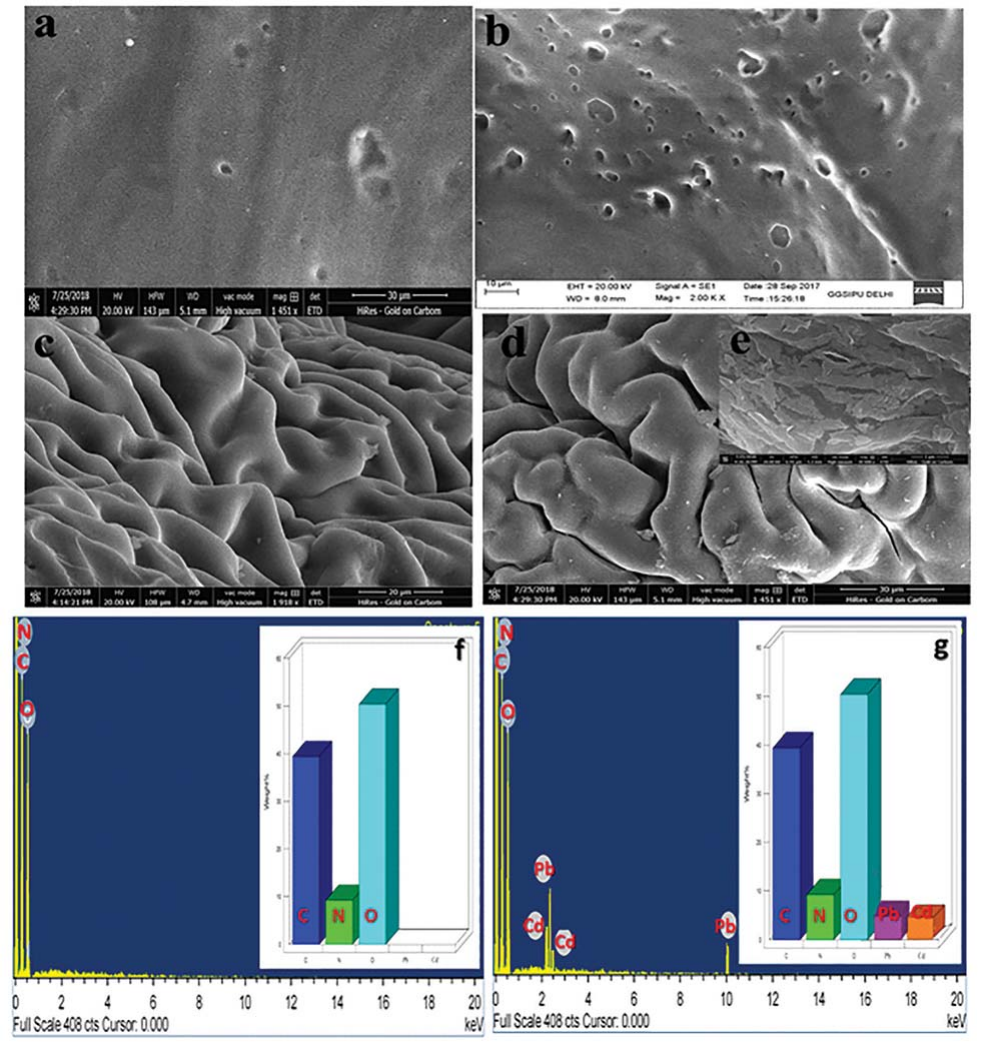
SEM micrographs of (a) CS, (b) CM, (c) CS-ag-CM, (d) Pb(II)/Cd(II)-CS-ag-CM(e) Pb(II)/Cd(II)-CS-ag-CM and EDX spectrum of (f) CS-ag-CM and (g) Pb(II)/Cd(II)-CS-ag-CM.

The Royal Society of Chemistry apologises for these errors and any consequent inconvenience to authors and readers.

## Supplementary Material

